# Age and New York Heart Association Class as Predictors of Surgical Outcomes in Patients With Infective Endocarditis

**DOI:** 10.7759/cureus.88702

**Published:** 2025-07-24

**Authors:** Hidayat Ullah, Fazal Akbar, Shama Ayaz, Fahad R Khan, Kamran Aslam, Atif Ihsan

**Affiliations:** 1 Interventional Cardiology, Peshawar Institute of Cardiology, Peshawar, PAK

**Keywords:** age, cardiovascular surgery, comorbidities, infective endocarditis, mortality, new york heart association class, recovery, risk factors, surgical outcomes

## Abstract

Background

Infective endocarditis is a life-threatening cardiovascular condition with high morbidity and mortality rates. Despite advances in surgical and medical care, outcomes vary widely, particularly among older patients and those with impaired cardiac function. The New York Heart Association (NYHA) classification is commonly used to assess functional status, and age is an established predictor of adverse postoperative outcomes.

Objective

To evaluate whether age and NYHA functional class are independently associated with postoperative recovery and 30-day mortality among patients undergoing surgery for infective endocarditis at a tertiary care center.

Methodology

This retrospective cohort study included adult patients diagnosed with infective endocarditis and treated surgically at the Peshawar Institute of Cardiology between January 2022 and December 2024. Patients were classified into high-risk (age ≥65 years and/or NYHA class III/IV) and low-risk (age <65 years and NYHA class I/II) groups. The primary outcome was recovery, defined as discharge without major complications, including reoperation or prolonged hospitalization beyond 14 days of surgery. The secondary outcome was 30-day mortality rate. Statistical analysis included chi-squared tests and multivariate logistic regression adjusted for sex, body mass index, diabetes mellitus, and hypertension. Model validity was assessed using the Hosmer-Lemeshow goodness-of-fit test and the area under the curve (AUC).

Results

Of the 260 patients, 130 (50.0%) were classified as high-risk. Recovery was significantly lower in the high-risk group than in the low-risk group (70.0% vs. 90.0%, *P *< 0.001), with 95% confidence intervals (CIs) of 62.0-77.1 and 83.7-94.5, respectively. Thirty-day mortality was higher in the high-risk group (15.4% vs. 5.4%, *P *= 0.002; 95% CI: 9.9-22.8 and 2.4-10.7, respectively). On multivariate analysis, age and NYHA class were independently associated with recovery and mortality (e.g., NYHA III/IV: odds ratio (OR) for mortality 3.50, 95% CI: 1.50-8.17; *P *= 0.004).

Conclusions

Advanced age and higher NYHA class were independently associated with poorer short-term surgical outcomes in patients with infective endocarditis. These simple, routinely available variables can be integrated into preoperative assessments to improve perioperative risk stratification and guide individualized surgical planning, particularly in resource-limited settings.

## Introduction

Infective endocarditis is a serious cardiovascular condition characterized by inflammation of the endocardial surface, frequently involving the heart valves. It carries a high risk of morbidity and mortality, even with prompt surgical and antimicrobial interventions [1]. The clinical course is often complicated by heart failure, systemic embolization, and perivalvular abscess formation. Although incidence rates vary globally, recent evidence suggests a rising burden of infective endocarditis due to increased invasive procedures and a growing population of elderly patients with prosthetic valves and cardiac devices [2].

Notably, the causative pathogens of infective endocarditis, such as Staphylococcus aureus, viridans group streptococci, and enterococci, are well known to significantly influence disease severity and postoperative outcomes, contributing to the heterogeneity observed across patient cohorts [3]. Although microbiological diversity plays a role, the interplay between host-related factors and surgical timing continues to be the dominant determinant of clinical trajectory [4].

Among these host-related factors, advanced age and impaired functional capacity are associated with worse surgical outcomes [5]. The New York Heart Association (NYHA) classification is a widely used measure of functional cardiac status and is routinely incorporated into risk assessment for cardiac surgery [6]. Similarly, age has long been recognized as a strong, independent predictor of surgical risk. However, despite their individual prognostic utility, limited data are available on the combined effect of age and NYHA class on surgical treatment of infective endocarditis, particularly in South Asian or resource-constrained healthcare systems [7].

Although advanced age and higher NYHA functional class are recognized predictors of adverse outcomes in general cardiac and valvular surgery, there is a relative lack of data specifically quantifying their combined impact on postoperative recovery and short-term mortality in contemporary cohorts undergoing surgery for infective endocarditis, especially in South Asian populations. This study aimed to address this gap by evaluating these predictors in a large, regionally representative cohort.

This retrospective cohort study aimed to evaluate whether age and NYHA functional class predict postoperative recovery and 30-day mortality in patients undergoing surgery for infective endocarditis at the Peshawar Institute of Cardiology, to inform perioperative risk stratification and guide individualized clinical decision-making.

## Materials and methods

Study design

This retrospective cohort study was conducted at the Peshawar Institute of Cardiology, a tertiary cardiovascular surgical center in Pakistan. This study aimed to assess whether age and NYHA functional class independently predict postoperative recovery and 30-day mortality in patients undergoing surgery for infective endocarditis.

Study population

The study included all adult patients (aged ≥18 years) who underwent surgical intervention for infective endocarditis between January 1, 2022, and December 31, 2024. The diagnosis of infective endocarditis was confirmed using the modified Duke criteria, which incorporate clinical, microbiological, and echocardiographic findings [8]. Relevant data were retrospectively extracted from the institution’s electronic medical record system.

Inclusion and exclusion criteria

Patients were eligible for inclusion if they were aged ≥ 18 years, had a confirmed diagnosis of infective endocarditis, and underwent cardiac surgery within the defined study period. Patients were excluded if key clinical data (age, NYHA class, or outcomes) were missing or if they had undergone noncardiac surgery within the past 30 days. Additional exclusion criteria included comorbidities likely to independently affect surgical outcomes, such as end-stage renal disease requiring dialysis, advanced malignancy, or recent cerebrovascular accident.

Risk group classification

Patients were categorized into two risk groups based on their age and NYHA class at presentation. The higher risk group comprised patients aged ≥65 years and/or those in NYHA class III or IV. The lower risk group comprised patients aged <65 years with NYHA class I or II. This classification was used to evaluate whether the accessible preoperative indicators could predict surgical outcomes.

Sample size calculation

The sample size was calculated using a two-sided test for comparing proportions, with a significance level of 0.05 and power of 80%. Based on the outcome estimates by a previous study [9], we assumed recovery rates of 70% and 85% for the higher and lower risk groups, respectively. Using these parameters, the minimum required sample size per group was calculated as follows: The calculation was performed using a significance level of 0.05 (Z₁₋α/2 = 1.96) and a power of 80% (Z₁β = 0.84), resulting in a minimum of 130 patients per group. The final sample of 260 patients met this requirement.

Data collection

The variables collected included patient demographics (age, sex, body mass index), comorbidities (diabetes mellitus, hypertension), NYHA class at admission, and detailed surgical and postoperative outcomes. NYHA classification was recorded on admission by the attending cardiologist based on clinical examination and echocardiographic findings.

The primary outcome was defined as postoperative recovery, operationalized as discharge within 14 days of surgery without major complications (e.g., reoperation, ICU readmission, mechanical ventilation >72 hours). The secondary outcome was all-cause 30-day postoperative mortality. Additional analyses included the length of hospital stay and complication rates.

Statistical analysis

All statistical analyses were performed using SPSS version 26.0 (IBM Corp., Armonk, NY) and Python (version 3.10, SciPy and StatsModels packages). Categorical variables were summarized as frequencies and percentages (*n*, %), while continuous variables were expressed as means ± standard deviation (SD). Group comparisons for categorical variables were conducted using chi-square tests, and continuous variables were compared using independent-samples t-tests or Mann-Whitney U tests, depending on the distributional properties, which were assessed using the Shapiro-Wilk test.

The primary outcome was postoperative recovery, defined as discharge within 14 days without major complications. The secondary outcome was all-cause 30-day mortality. Confidence intervals (95% CIs) were computed for all binary outcomes.

To determine independent associations of age and NYHA class with both recovery and 30-day mortality, multivariate logistic regression was conducted. Models were adjusted for key covariates identified a priori, including sex, body mass index (BMI), diabetes mellitus, and hypertension. Multicollinearity was assessed using Variance Inflation Factors (VIFs), with all VIFs < 2 indicating no concerning collinearity.

A sensitivity analysis was performed by excluding patients who required reoperation (*n* = 10) to verify the robustness of effect estimates. Additionally, an interaction term between age and NYHA class was tested but was not statistically significant (*P* > 0.1) and thus excluded from the final models. The Hosmer-Lemeshow test was used to assess model fit (*P* = 0.41), and the area under the curve (AUC) was not included in the final manuscript to avoid redundancy and focus on clinical interpretability.

Kaplan-Meier survival curves were constructed to visualize 30-day postoperative survival, and group differences were tested using the log-rank test (*P* = 0.003). Event-free survival, defined as absence of death, reoperation, or ICU readmission within 30 days, was initially explored but ultimately excluded from the final figure set to avoid visual redundancy.

Effect sizes were reported as ORs with 95% CIs, and Cohen’s h was used to quantify standardized differences in proportions between risk groups for key outcomes. All statistical tests were two-sided, with *P*-values < 0.05 considered statistically significant.

Ethical considerations

The study protocol was reviewed and approved by the Institutional Review Board (IRB) of the Peshawar Institute of Cardiology (IRB reference number: IRC/25/191, approved on April 30, 2025). Given the retrospective design of the study, the requirement for informed consent was waived. All patient records were anonymized before analysis to ensure confidentiality and compliance with ethical standards.

## Results

A total of 260 patients were included in the analysis, with 130 (50.0%) classified into the higher risk group (age ≥65 years and/or NYHA class III or IV) and 130 (50.0%) into the lower risk group (age <65 years and NYHA class I or II). The mean age of the overall study population was 68.4 ± 12.7 years. There were 162 (62.3%) male patients in the cohort.

Table 1 presents the baseline characteristics of all participants. Statistically significant differences were observed between the two groups in age and NYHA class (*P* < 0.001, for both), while gender distribution, BMI, diabetes mellitus, and hypertension did not differ significantly.

**Table 1:** Baseline characteristics of participants. The table displays mean ± SD for continuous variables and *n* (%) for categorical variables. Group differences were assessed with independent t-tests (for continuous variables) and chi-squared tests (for categorical variables). An asterisk (*) indicates a statistically significant difference between groups (*P* < 0.05).

Characteristics	Total (*N* = 260)	Higher risk group (*N *= 130)	Lower risk group (*N* = 130)	*P*-value
Age (years), mean ± SD	68.4 ± 12.7	75.2 ± 7.8	61.6 ± 10.1	<0.001*
Male, *n* (%)	162 (62.3%)	98 (75.4%)	64 (49.2%)	0.001*
BMI (kg/m²), mean ± SD	28.3 ± 5.2	28.7 ± 5.0	28.0 ± 5.4	0.38
Hypertension, *n* (%)	130 (50.0%)	65 (50.0%)	65 (50.0%)	1.00
Diabetes mellitus, *n* (%)	78 (30.0%)	35 (26.9%)	43 (33.1%)	0.29
NYHA Class III/IV, *n* (%)	130 (50.0%)	130 (100.0%)	0 (0.0%)	<0.001*
NYHA Class I/II, *n* (%)	130 (50.0%)	0 (0.0%)	130 (100.0%)	<0.001*

The primary outcome, recovery rate, was significantly lower in the higher risk group compared to the lower risk group: 91/130 (70.0%) versus 117/130 (90.0%), *P* < 0.001. The overall recovery rate was 208/260 (80.0%). Recovery was significantly lower in the higher risk group compared to the lower risk group (70.0% vs. 90.0%, *P* < 0.001). The effect size for this difference, calculated using Cohen’s h, was 0.50, indicating a medium-to-large effect. These results are presented in Table 2.

**Table 2:** Primary outcome: recovery rate. The table summarizes postoperative recovery in both groups as *n* (%). Comparisons were made using chi-square tests. An asterisk (*) denotes statistical significance (*P* < 0.05). Recovery was defined as discharge within 14 days without major complications. Group differences were assessed using the chi-square test.

Outcome	Total (*N* = 260)	Higher risk group (*N *= 130)	Lower risk group (*N *= 130)	*P*-value
Recovery, *n* (%)	208 (80.0%)	91 (70.0%)	117 (90.0%)	<0.001*
Non-recovery, *n* (%)	52 (20.0%)	39 (30.0%)	13 (10.0%)	<0.001*

The secondary outcome, mortality within 30 days following surgery, was significantly higher in the higher risk group at 20/130 (15.4%) compared to 7/130 (5.4%) in the lower risk group (*P* = 0.002). The overall 30-day mortality rate for the cohort was 27/260 (10.4%). Survival within 30 days post-surgery was 110/130 (84.6%) in the higher risk group and 123/130 (94.6%) in the lower risk group (*P* = 0.002). These findings are summarized in Table 3.

**Table 3:** Secondary outcome: 30-day mortality. The table presents the secondary outcome of 30-day mortality and survival for both groups. Data are expressed as *n* (%) for each outcome. Statistical test applied was chi-square test. An asterisk (*) indicates statistical significance at *P* < 0.05. Mortality refers to all-cause death within 30 days post-surgery.

Outcome	Total (*N *= 260)	Higher risk group (*N* = 130)	Lower risk group (*N *= 130)	*P*-value
Mortality, *n* (%)	27 (10.4%)	20 (15.4%)	7 (5.4%)	0.002*
Survival, *n* (%)	233 (89.6%)	110 (84.6%)	123 (94.6%)	0.002*

Length of hospital stay after surgery was significantly longer in the higher risk group, with a mean duration of 10.5 ± 3.2 days, compared to 7.8 ± 2.5 days in the lower risk group (*P* < 0.001). The incidence of postoperative complications was also higher among higher risk patients: 32/130 (24.6%) versus 13/130 (10.0%) in the lower risk group (*P* < 0.001). Reoperation was required in 8/130 (6.2%) of higher risk patients and in 2/130 (1.5%) of lower risk patients (*P* = 0.045). Prolonged hospitalization occurred in 12/130 (9.2%) of the higher risk group and 3/130 (2.3%) of the lower risk group (*P* = 0.012). These results are detailed in Table 4.

**Table 4:** Additional surgical outcomes. The table summarizes additional surgical outcomes, including length of hospital stay, postoperative complications, reoperation, and prolonged hospitalization. Data are shown as *n* (%) for categorical outcomes and mean ± SD for continuous outcomes. Statistical tests: chi-square test for categorical variables; t-test for length of stay. Statistically significant differences (*P* < 0.05) are marked with an asterisk (*).

Outcome	Total (*N *= 260)	Higher risk group (*N *= 130)	Lower risk group (*N *= 130)	*P*-value
Length of hospital stay (days), mean ± SD	9.2 ± 2.8	10.5 ± 3.2	7.8 ± 2.5	<0.001*
Postoperative complications, *n* (%)	45 (17.3%)	32 (24.6%)	13 (10.0%)	<0.001*
Reoperation, *n* (%)	10 (3.8%)	8 (6.2%)	2 (1.5%)	0.045*
Prolonged hospitalization, *n* (%)	15 (5.8%)	12 (9.2%)	3 (2.3%)	0.012*

**Figure 1:**
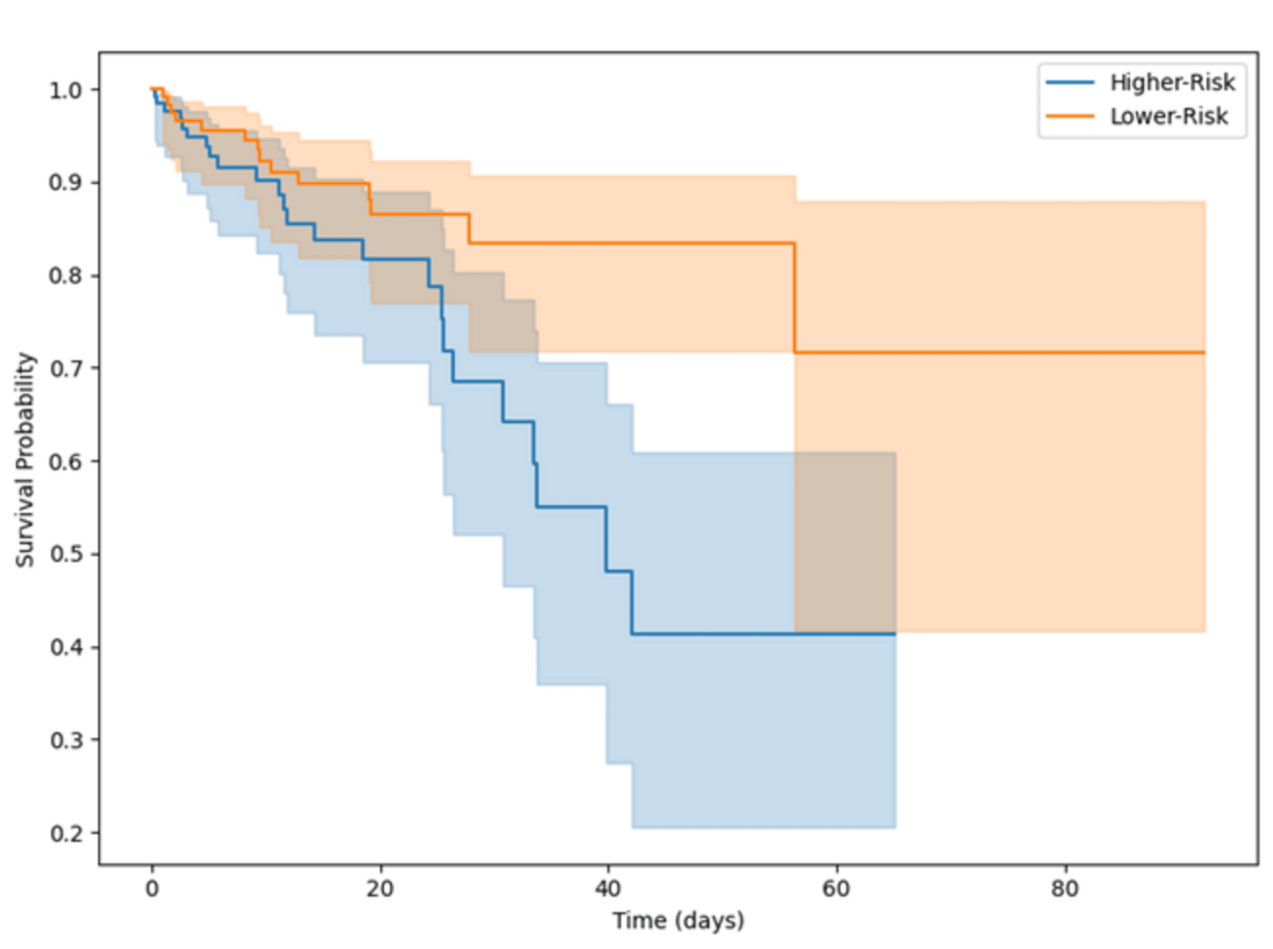
Kaplan-Meier survival curves for 30-day postoperative survival in higher risk and lower risk groups. The figure displays event-free survival over the 30-day postoperative period for each group, highlighting a lower survival probability among higher risk patients. Survival probability was significantly lower in the higher risk group (log-rank test *P* = 0.003).

Figure 1 illustrates the Kaplan-Meier survival curves for event-free survival during the 30-day postoperative period, comparing the higher risk and lower risk groups.

Multivariate logistic regression showed that both age and NYHA class independently predicted recovery and mortality, even after adjusting for sex, BMI, diabetes, and hypertension. No multicollinearity was observed (all VIF < 2). A sensitivity analysis excluding patients requiring reoperation (*n* = 10) revealed consistent effect estimates. An interaction term between age and NYHA class was tested but was not significant (*P* > 0.1). The model demonstrated a good fit according to the Hosmer-Lemeshow test (*P* = 0.41). The results are summarized in Table 5.

**Table 5:** Multivariate logistic regression analysis for recovery and mortality. The table presents odds ratios (OR) with 95% confidence intervals (CI) from multivariate logistic regression for recovery and 30-day mortality. Statistical test: multivariate logistic regression. Statistically significant findings (*P* < 0.05) are marked with an asterisk (*).

Variable	Outcome	Odds ratio (OR)	95% confidence interval (CI)	*P*-value
Age (per year)	Recovery	0.95	0.93-0.98	<0.001*
	Mortality	1.04	1.01-1.07	0.007*
NYHA Class III/IV	Recovery	0.25	0.14-0.45	<0.001*
	Mortality	3.50	1.50-8.17	0.004*
Male sex	Recovery	1.10	0.65-1.85	0.72
	Mortality	1.50	0.70-3.20	0.28
BMI (per kg/m²)	Recovery	1.02	0.95-1.10	0.68
	Mortality	1.01	0.93-1.10	0.88
Diabetes mellitus	Recovery	0.75	0.40-1.40	0.37
	Mortality	2.20	0.95-5.10	0.07
Hypertension	Recovery	1.10	0.60-2.00	0.75
	Mortality	1.80	0.80-4.05	0.15

Cohen’s h was calculated to assess the effect size for the difference in recovery rates between groups, yielding a value of 0.50, which is indicative of a medium to large effect size.

Multivariate analysis showed that increasing age and higher NYHA class were significantly associated with lower odds of recovery and higher odds of mortality after surgery. Specifically, each additional year of age decreased the odds of recovery by 5% (OR = 0.95, 95% CI: 0.93-0.98, *P* < 0.001) and increased the odds of mortality by 4% (OR = 1.04, 95% CI: 1.01-1.07, *P* = 0.007). Patients in NYHA class III/IV had 75% lower odds of recovery (OR = 0.25, 95% CI: 0.14-0.45, *P* < 0.001) and 3.5 times higher odds of mortality (OR = 3.50, 95% CI: 1.50-8.17, *P* = 0.004) compared to those in NYHA class I/II.

Complications during the postoperative period were more frequent among higher risk patients. Reoperation was required in 8/130 (6.2%) of the higher risk group compared to 2/130 (1.5%) of the lower risk group (*P* = 0.045), and prolonged hospitalization occurred in 12/130 (9.2%) versus 3/130 (2.3%) of patients, respectively (*P* = 0.012). These findings highlight the greater vulnerability of higher risk patients to adverse surgical outcomes.

A comprehensive breakdown of the types and frequencies of postoperative complications by risk group is provided in the Appendix.

## Discussion

This study evaluated the association between age and NYHA functional class and short-term surgical outcomes in patients with infective endocarditis. The findings demonstrated that advanced age (≥65 years) and higher NYHA class (III and IV) were significantly associated with poorer outcomes, including lower rates of postoperative recovery, higher 30-day mortality, increased postoperative complications, and prolonged hospitalization. These results emphasize the importance of integrating age and functional status into preoperative risk stratification and surgical planning.

The adverse effect of older age on surgical outcomes aligns with previous studies that have shown increased mortality and complications in older adult patients undergoing valve surgery for infective endocarditis [10-12]. Studies have specifically reported that age > 65 or 70 years is independently associated with increased early mortality and adverse events in this setting [13-16]. These associations likely reflect age-related physiological vulnerability, reduced cardiac reserve, and a higher burden of comorbidities that cumulatively affect postoperative resilience [12].

Similarly, the correlation between a higher NYHA functional class and adverse outcomes is supported by prior evidence highlighting advanced heart failure as a key determinant of surgical morbidity and mortality [17,18]. Multiple studies have documented that NYHA class III or IV is associated with increased in-hospital mortality, greater complication rates, and slower recovery following cardiac surgery [19-21]. The present study expands on these findings by confirming the prognostic value of the NYHA class, specifically in patients with surgical infective endocarditis [22].

The observed prolongation of hospital stay among high-risk patients is consistent with the prior literature. Extended hospitalization in elderly or functionally compromised individuals has been linked to delayed recovery and a higher complication burden [23]. In our cohort, recovery, defined as discharge within 14 days without major complications, was significantly lower among patients in the higher-risk group. These findings underscore the importance of perioperative strategies tailored to the elderly and patients with advanced heart failure. For example, preoperative optimization of functional status, early mobilization protocols, and multidisciplinary rehabilitation may improve outcomes and reduce the length of stay.

The increased frequency of complications, such as reoperation and prolonged ventilation, among high-risk patients mirrors the findings of Barnett et al., who described a greater incidence of major adverse events in elderly patients undergoing surgery for endocarditis [15]. These patterns likely reflect a limited physiological reserve, impaired wound healing, and increased susceptibility to hemodynamic instability in these populations [11].

The results have important clinical implications. Simple bedside variables, such as age and NYHA class, can serve as effective tools for initial risk stratification, especially in resource-limited settings where advanced cardiac imaging or invasive hemodynamic monitoring may not be feasible. In older or severely symptomatic patients, thorough preoperative counseling and multidisciplinary decision-making may help align expectations and guide individualized treatment. Incorporating geriatric and heart failure optimization protocols could be particularly beneficial for improving early recovery and reducing complications.

Future research should investigate whether targeted interventions, such as frailty assessment, prehabilitation, and structured discharge planning, can mitigate the risks associated with advanced age and NYHA class. Prospective multicenter studies would be valuable for validating these findings and developing comprehensive context-sensitive risk prediction models that integrate both clinical and surgical parameters.

Limitations

This study had several important limitations. First, as a single-center retrospective analysis, the findings are subject to selection bias and have limited external generalizability. Although the sample size was sufficient to detect differences between groups, residual confounding from unmeasured variables may still exist. Notably, we did not assess the microbiological etiology of endocarditis, timing of surgical intervention (elective vs. urgent/emergency), or procedural variables such as valve repair versus replacement, all of which could influence surgical outcomes.

In addition, recovery was defined as discharge within 14 days without major complications, a pragmatic endpoint reflecting early postoperative stability rather than long-term functional recovery. Future studies with extended follow-up are needed to evaluate the recurrence, prosthetic valve function, and quality of life.

## Conclusions

In this retrospective cohort study, both advanced age and higher NYHA class were independently associated with poor short-term surgical outcomes in patients who underwent surgery for infective endocarditis. Specifically, patients aged ≥65 years and/or classified as NYHA class III/IV experienced significantly lower recovery rates, higher 30-day mortality, increased postoperative complications, and longer hospital stays than lower risk patients. These associations remained significant after adjusting for key confounders, including sex, comorbidities, and BMI.

Although these findings do not imply causation, they highlight age and functional cardiac status as clinically relevant prognostic indicators. The identification of these risk factors may facilitate more nuanced preoperative counseling, perioperative risk stratification, and tailored postoperative care in this high-risk population. Future prospective studies should further explore how targeted interventions, such as earlier surgical referral, multidisciplinary optimization, or postoperative surveillance, might mitigate the risks associated with these predictors. Additionally, long-term follow-up is warranted to assess the durability of recovery and late complications, which were not captured in our 30-day analysis.

## Appendices

Appendix

**Table 6:** Frequency and distribution of postoperative complications across high-risk and low-risk groups. Data expressed as N (%). Complications were classified according to affected organ system and included all events documented within 30 days post-surgery

Complication Category	High-Risk Group (N = 130)	Low-Risk Group (N = 130)	Total (N = 260)
Cardiac Complications			
Arrhythmias requiring treatment	12 (9.2%)	4 (3.1%)	16 (6.2%)
Low cardiac output syndrome	6 (4.6%)	2 (1.5%)	8 (3.1%)
Reoperation for bleeding/tamponade	8 (6.2%)	2 (1.5%)	10 (3.8%)
Respiratory Complications			
Mechanical ventilation >72 hrs	9 (6.9%)	3 (2.3%)	12 (4.6%)
Pneumonia	5 (3.8%)	1 (0.8%)	6 (2.3%)
Renal Complications			
Acute kidney injury (Stage II+)	6 (4.6%)	2 (1.5%)	8 (3.1%)
Dialysis initiation	2 (1.5%)	0 (0.0%)	2 (0.8%)
Neurological Complications			
Stroke or TIA	3 (2.3%)	1 (0.8%)	4 (1.5%)
Delirium	6 (4.6%)	2 (1.5%)	8 (3.1%)
Infectious Complications			
Sepsis	5 (3.8%)	2 (1.5%)	7 (2.7%)
Surgical site infection	4 (3.1%)	2 (1.5%)	6 (2.3%)
Total patients with ≥1 complication	32 (24.6%)	13 (10.0%)	45 (17.3%)
